# In vitro induction of hair follicle signatures using human dermal papilla cells encapsulated in fibrin microgels

**DOI:** 10.1111/cpr.13528

**Published:** 2023-08-04

**Authors:** Cristina Quílez, Leticia Valencia, Jorge González‐Rico, Leticia Suárez‐Cabrera, Lidia Amigo‐Morán, José Luis Jorcano, Diego Velasco

**Affiliations:** ^1^ Department of Bioengineering Universidad Carlos III de Madrid Leganés Spain; ^2^ Fundación Instituto de Investigación Sanitaria de la Fundación Jiménez Díaz Madrid Spain; ^3^ Department of Continuum Mechanics and Structural Analysis Universidad Carlos III de Madrid Leganés Spain; ^4^ Instituto De Investigacion Sanitaria Gregorio Marañon Madrid Spain

## Abstract

Cellular spheroids have been described as an appropriate culture system to restore human follicle dermal papilla cells (hFDPc) intrinsic properties; however, they show a low and variable efficiency to promote complete hair follicle formation in in vivo experiments. In this work, a conscientious analysis revealed a 25% cell viability in the surface of the dermal papilla spheroid (DPS) for all culture conditions, questioning whether it is an appropriate culture system for hFDPc. To overcome this problem, we propose the use of human blood plasma for the generation of fibrin microgels (FM) with encapsulated hFDPc to restore its inductive signature, either in the presence or in the absence of blood platelets. FM showed a morphology and extracellular matrix composition similar to the native dermal papilla, including Versican and Collagen IV and increasing cell viability up to 85%. While both systems induce epidermal invaginations expressing hair‐specific keratins K14, K15, K71, and K75 in in vitro skin cultures, the number of generated structures increases from 17% to 49% when DPS and FM were used, respectively. These data show the potential of our experimental setting for in vitro hair follicle neogenesis with wild adult hFDPc using FM, being a crucial step in the pursuit of human hair follicle regeneration therapies.

## 1 BACKGROUND

Dermal papilla (DP) is a cell aggregate composed of specialized fibroblasts embedded in a proteoglycan‐rich extracellular matrix involved in hair follicle induction and maintenance during its entire lifetime.^1^, ^2^, ^3^, ^4^ After extraction and culture, if human follicle dermal papilla cells (hFDPc) are cultured in the appropriate three‐dimensional (3D) environment, such as spheroids, they can restore their native signature and induce hair follicle formation in mice.^5^

Although cellular spheroids are probably the most popular 3D culture system to mimic cell native microenvironment,^6^, ^7^, ^8^ they face issues associated with poor cell viability.^9^, ^10^ Besides, they do not replicate cell‐to‐matrix interaction present in most of the 3D microenvironment of the body.^11^ On this basis, biomaterials‐assisted spheroid engineering has been extensively explored for regenerative purposes,^12^, ^13^, ^14^ better mimicking the 3D microenvironment and enhancing cell survival.^15^ While biomaterials such as hyaluronic acid and collagen have been used to improve hFDPc culture in vitro,^16^, ^17^, ^18^ they do not successfully mimic the extracellular matrix heterogeneity of the native human DP,^4^ which compromises the underpinning features of DP structure.^19^, ^20^, ^21^

Due to its unique composition,^22^ human blood plasma has emerged as a new material of interest widely used in the treatment of hair loss, hFDPc culture, and hair follicle induction.^23^, ^24^, ^25^ In addition to this, the use of human blood plasma as a source of human fibrin for autologous skin replacement has shown the ability of embedded fibroblasts to produce natural collagen.^26^, ^27^ Based on this principle and in the extensive experience of our group working with bilayer plasma‐derived skin equivalents,^28^, ^29^, ^30^ human blood plasma arises as a potential candidate for hFDPc encapsulation to mimic human DP microenvironment.

## 2 QUESTIONS ADDRESSED

The goal of this study is to investigate whether hFDPc cell encapsulation in plasma‐derived fibrin microgels (FM) is a feasible alternative culture system for dermal papilla spheroids (DPS). Both systems will be characterized in terms of morphology, cell viability, restoration of its signature and ability to induce hair follicle cell differentiation.

## 3 EXPERIMENTAL DESIGN

### 3.1 DPS and FM culture

For spheroid and microgel generation, primary hFDPc (C‐12071; PromoCell) with less than six population doublings were used. DPS were cultured according to a previously described method^31^, ^32^ (Figure S1A). For dynamic culture conditions, DPS were generated and cultured using a nutator.^33^ FM at a final fibrin concentration of 2.4 mg/mL were generated based on the previously described methods by the deposition of 2 μL drops of working solution onto the lid of a 35‐mm culture plate and incubated for 15 min at 37°C and 5% CO_2_ ^30^ (Text S1). Finally, FM were transferred to a low adherence plate and cultured for 48 h before use (Figure S1B). To inspect the effect of blood platelets in FM cell culture, platelet poor/rich human blood plasma (PPP/PRP) donated by CCST blood bank was used for the generation of FM. To analyse the effect of different densities,^34^ both DPS and FM were generated using a final number of 750, 1500, 3000, and 6000 cells.

### 3.2 Morphometric, viability, and proliferation analysis

DPS and FM size and morphology were studied by analysing spheroid diameter in a self‐designed programme in MATLAB (MathWorks) using images from an inverted microscope. The area was calculated as the projected area of the spheroid ($A_{S}$) in μm^2^. Cell‐mediated contraction of FM was measured by area evolution through time and expressed in terms of the swelling ratio $\left({\mathrm{SR}}_{A}\right)$.^35^ In addition, cell distribution and morphology within the FM were inspected using CellTracker™ Orange Dye (C34551; Thermo Fisher Scientific) after 48 h in culture (D_0_). Dermal papilla cell viability and proliferation within DPS and FM were analysed using three different methods: Live/Dead® Viability assay for mammalian cells, by the incorporation of thymidine analogue BrdU (Thermo Fisher Scientific) and CellTiter‐Glo® 3D Cell Viability Assay following manufacturer's instruction.

### 3.3 In vitro hair follicle cell differentiation

To induce in vitro hair follicle cell differentiation plasma‐derived matrices were prepared based on the previously described methods^30^, ^36^ at a final fibrin concentration of 2.4 mg/mL using both PPP and PRP (Figure S1C; Text S1; Figure S2). The re‐storage of the dermal papilla cell signature was proven by the expression of well‐known proteins by conventional immunocytochemistry at D_0_ and D_15_ (Table S1)_._ Besides, protein expression was also characterized through real‐time quantitative PCR (RT‐qPCR) at D_0_ (Text S2; Table S2). In addition, hair follicle cell differentiation was tracked with haematoxylin and eosin tinction, and immunofluorescence of well‐known proteins present in the hair follicle (Table S1). Efficiency $\left(E_{i}\right)$ of each culture system to induce hair follicle cell differentiation was expressed as an approximation of the total number of structures expressing hair follicle proteins $\left(I_{r}\right)$ over the total number of seeded DPS/FM: $E_{i}\;\left(\%\right)=\left(I_{r}/I_{t}\right)\times 100.$

## 4 RESULTS

### 4.1 FM showed similar morphology to DPS and DP structure

**FIGURE 1:**
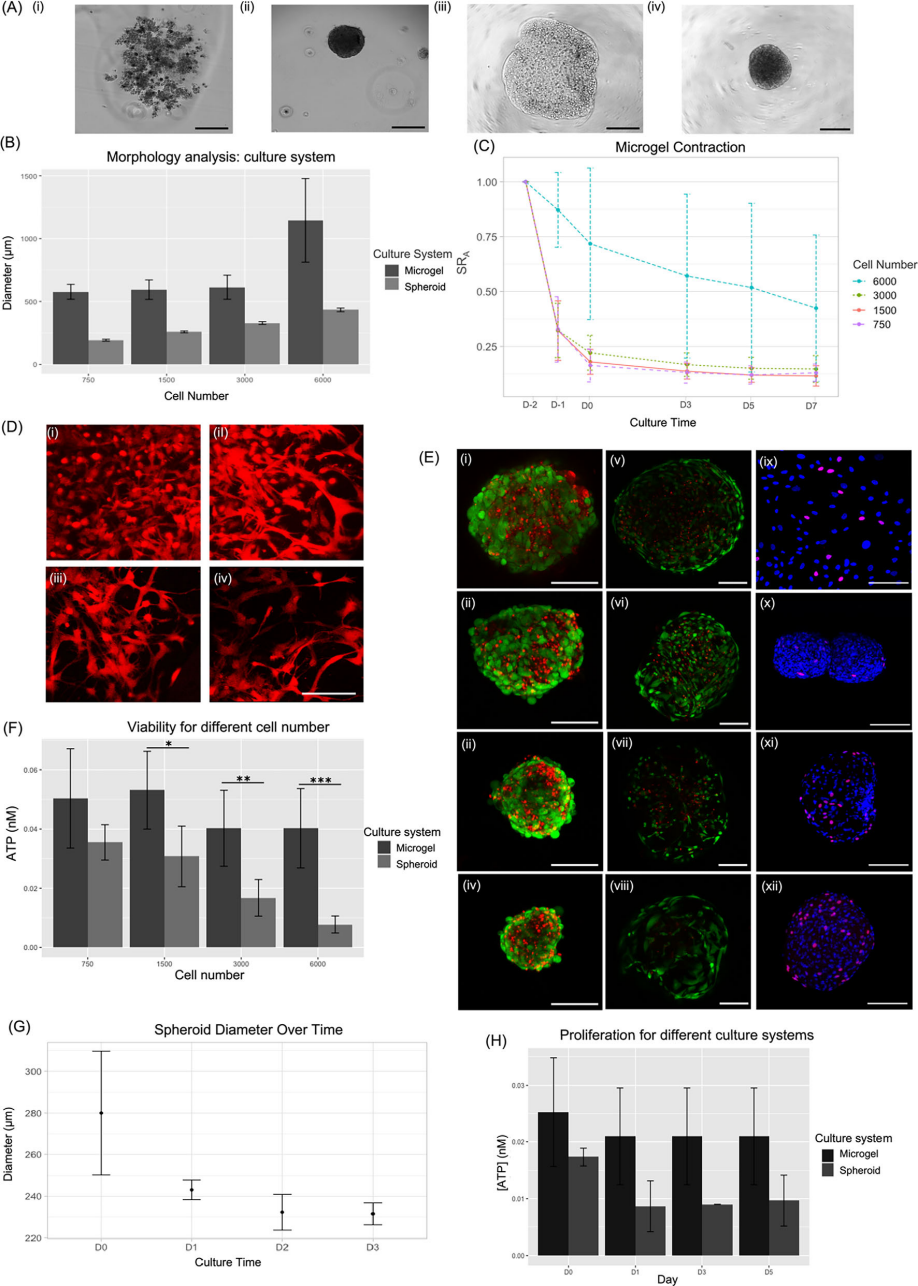
Morphometric, viability, and proliferation characterization of dermal papilla spheroids (DPS) and fibrin microgels (FM). (A) Visual inspection under the phase‐contrast microscope of: (i) human follicle dermal papilla cells (hFDPc) suspension, (ii) DPS, (iii) FM just after generation, and (iv) FM 48 h after generation. Scale bar = 200 μm. In (B) morphometric analysis of both DPS and FM for different cell numbers. (C) Size evolution of FM during 9 days after formation. In the *x*‐axis: culture time, (D_−2_); formation day, 1 day in culture (D_−1_); 2 days in culture (D_0_); 5 days in culture (D_3_); 7 days in culture (D_5_); and 10 days in culture (D_7_). Ten samples were analysed to extract morphology values. (D) Cell morphology using CellTracker™ Orange Dye (red fluorescence) inside the FM of different cell numbers after 48 h in culture: (i) 6000 cells; (ii) 3000 cells; (iii) 1500 cells; and (iv) 750 cells. Scale bar = 50 μm. (E) Viability of hFDPc in DPS (i–iv) and FM (v–vii) using Live/Dead® assay at D_0_ 6000 cells (i)18.3%‐ (v) 40.2% viability; 3000 cells (ii) 25.1%‐ (vi) 44.5% viability; 1500 cells (iii) 25.4%‐ (vii) 50% viability and 750 cells (iv) 23%‐ (viii) 85% viability. Proliferation, measured as BrdU incorporation, of hFDPc containing 3000 cells at D_0_: (ix) 2D culture (12% proliferation); (x) DPS (5% proliferation); (xi) PPP‐FM (16.7% proliferation); and (xii) PRP‐FM (16.7% proliferation). Viability (F) in terms of ATP at D_0_ of hFDPc for DPS and FM. (G) Size evolution of DPS during 5 days in culture after formation culture. Proliferation (H) in terms of ATP for FM and DPS containing 3000 cells. ATP concentration was normalized in terms of cell number. D_0_ was established 48 h after FM generation. In blue, cell nuclei stained with DAPI, in pink, BrdU‐positive cells. Three samples were analysed to extract cell viability and proliferation. Scale bar = 150 μm. Statistical significance: *p < 0.05; **p < 0.01; ***p < 0.001.

After 48 h in culture, both DPS and FM systems had a stable and circular shape DP structure (Figure 1A(ii, iv)). While DPS showed a diameter close to that of DP structure—280 μm—it increased by twofold for FM (Figure 1B). Moreover, FM contracted within the first 48 h up to 85% in the area (Figure 1C), a time at which hFDPc showed an elongated shape with branches characteristic of fibroblast‐like cells, particularly when they cleave to the extracellular matrix (Figure 1D). Nonetheless, as cell number increased some of them displayed a spherical morphology with very short branches (Figure 1D(i)) which correlates with less pronounced and more variable area reduction of FM (Figure 1C).

### 4.2 Cell viability increases in FM with respect to DPS

Cells withing DPS showed low cell viability—around 25%—with no significant differences for any of the culture conditions (cell number or static/dynamic culture), as confirmed by Live/Dead® assay (Figure 1E(i–iv); Table S3) and ATP expression (Figure S3). However, cells within FM showed viability values that range from 85% to 40%, corresponding to the lowest and the highest cell concentration (Figure 1E(v–viii)) and higher cell metabolic activity with respect to DPS (Figure 1F). Regarding cell proliferation, the progressive decrease of DPS size over time suggests that hFDPc do not proliferate within the spheroid (Figure 1G), as proved by metabolic activity of cells (Figure 1H). This was confirmed by BrdU tinction (Figure 1E(ix–xii)), which showed that at D_0_ the number of replicating cells increases from 5% to 16.7% in DPS and FM, respectively, regardless of the presence of blood platelets.

### 4.3 FM ensure restoration of hFDPc intrinsic properties

**FIGURE 2:**
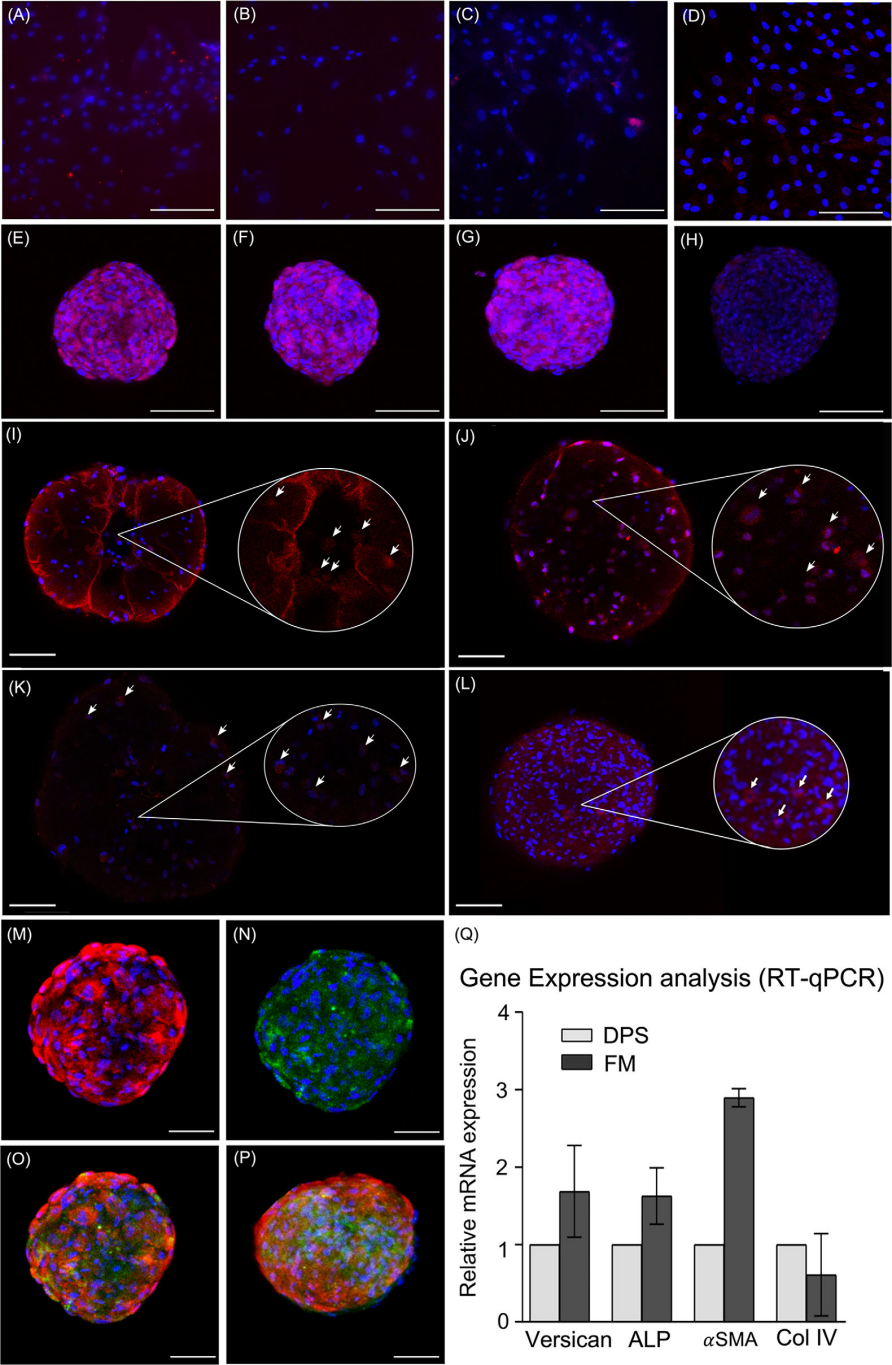
Protein expression characterization in dermal papilla spheroids (DPS) and fibrin microgels (FM). In red, immunofluorescence of human follicle dermal papilla cells (hFDPc) at D_0_ in 2D (A–C); DPS (D–H); and FM (I–L) containing 3000 cells for ALP (A, E, I); Versican (B, F, J); αSMA (C, G, K), and Col IV (D, H, L). In blue, cell nuclei stained with DAPI. D_0_ was established 48 h after FM generation. White arrows point to cell protein expression in a magnified view. Immunofluorescence of hFDPc inside FM containing 3000 cells at day D_15_ for (M) Versican and (N) Col IV. Merge images for (O) PPP‐FM and (P) PRP‐FM: in red, Versican, in green, Col IV. In blue, cell nuclei stained with DAPI. D_15_ was established 17 days after FM generation. Scale bar = 100 μm. Gene expression analysis of genes associated with hair follicle induction using RT‐qPCR (Q). Data were normalized to levels of the reference gene YWHAZ. Error bars represent the standard deviation calculated from two independent experiments for each condition.

Cell restoration of its intrinsic properties in both systems was proven by positive expression of ALP (Figure 2E,I), Versican (Figure 2F,J), and α‐SMA (Figure 2G,K) after 48 h in culture when compared to conventional 2D culture (Figure 2A–C), in both immunofluorescence and RT‐qPCR (Figure 2Q). Surprisingly, for immunofluorescence, Col IV expression was positive in cells within the FM (Figure 2L) and almost negligible in those of DPS (Figure 2H), whereas RT‐qPCR showed Col IV gene expression in both cases (Figure 2Q). In addition, cell‐mediated matrix remodelling was proven by positive expression of Col IV and Versican proteins at D_15_, regardless of the presence of blood platelets (Figure 2K,L).

### 4.4 FM system induce epidermal invaginations

**FIGURE 3:**
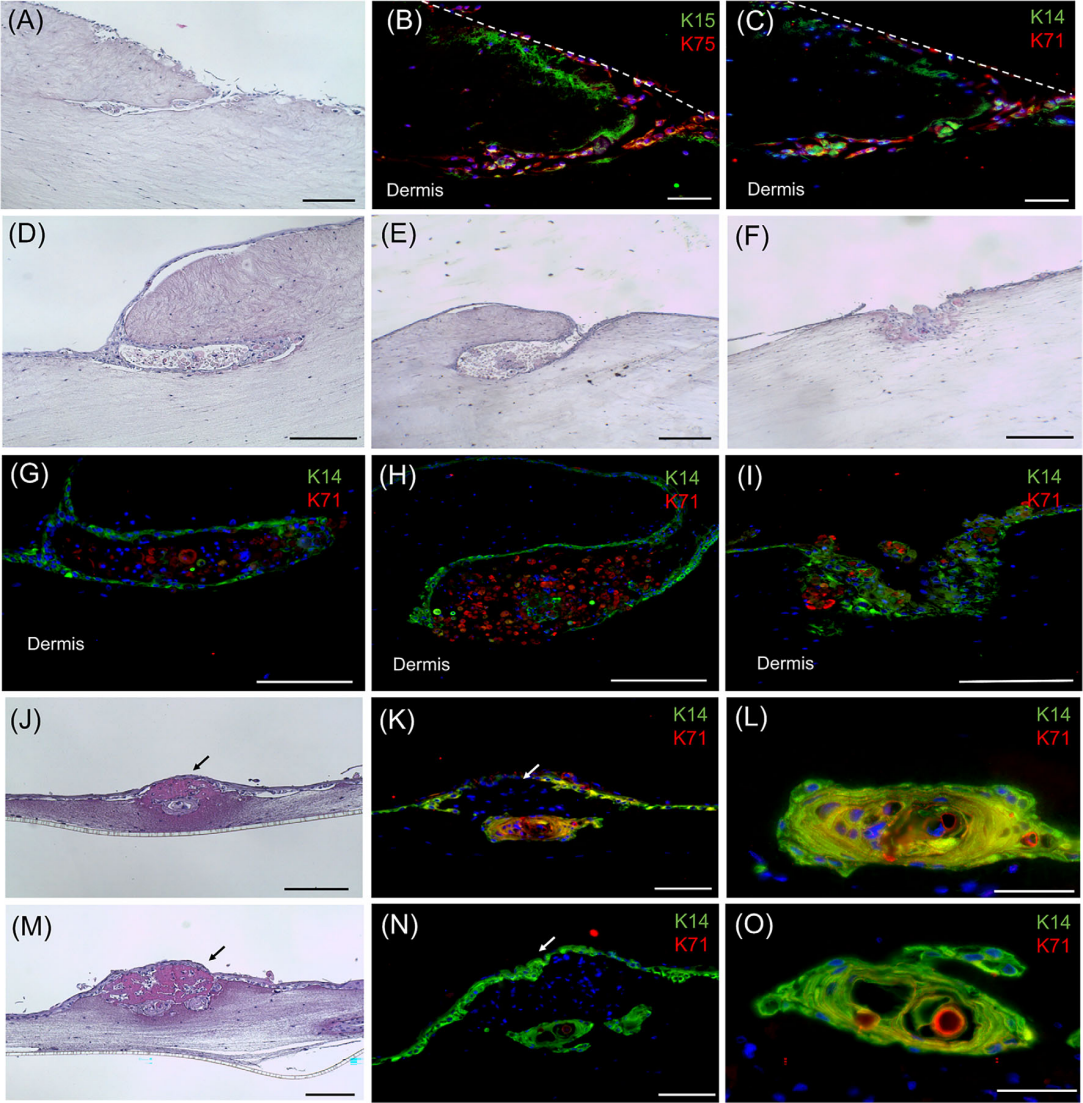
Epidermal invaginations expressing hair follicle signatures. Structures induced in organotypic skin cultures by dermal papilla spheroids after 3 weeks in culture (A–I): epidermal invagination structure (A, D, E,) expressing hair follicle specific keratins K15/K75 (B) and K14/K71 (C, G, H); induction of keratinocyte intussusception (F) expressing (I) K14/K71 hair follicle specific keratins. Structures induced in organotypic skin cultures by fibrin microgels (FM) after 3 weeks in culture (J–O): tubular structures (J, M) expressing hair follicle specific keratins K14/K71 (K, L, N, O). White and black arrows point to the FM in the dermal compartment of the construct. In blue, nuclei are stained with DAPI. Scale bar = 200 μm.

After 3 weeks in culture in in vitro skin equivalents, hFDPc showed positive expression of ALP, Versican, and Col IV proteins (Figure S4B–E) both in DPS and FM, proving the activity of hFDPc cells after prolonged culture times. Moreover, early signs of epidermal intussusception induced by DPS gave rise to structures of diverse morphologies (Figure 3A,D,E,F). Cell differentiation into hair follicle fate was proven by keratinocyte localized positive expression of hair germ K15, hair medulla K75 (Figure 3B), K14 for outer root sheath (ORS) structure and K71 for the inner root sheath (IRS) structure (Figure 3C,G,H,I). Alternatively, the characterization of organotypic constructs containing FM exhibited a very thin dermal component with nodules of approximately 400 μm at the surface, surrounded by a layer of keratinocytes (Figures 3J,M and S4F,H). They presented multilayered ellipsoidal structures of 100–200 μm diameter at the dermal interface with positive cell expression of ORS‐K14 and IRS‐K71 (Figures 3K,L,N,O and S4G,I), and no expression of hair germ K15 and hair medulla K75. Although both systems induced epidermal invaginations expressing hair follicle signatures, the DPS method showed a wide variability (8‐, 29%) between samples, with a mean efficiency of 17.4% whereas FM displayed similar efficiency values with a mean of 48.9%. Nonetheless, in both systems, no significant differences were found in the presence/absence of blood platelets.

## 5 CONCLUSION AND PERSPECTIVE

A hair follicle is a complex structure involved in key biological functions whose neogenesis is restricted to the embryo and cannot be regenerated during adulthood.^1^, ^37^, ^38^ Recently, two promising methodologies have successfully and efficiently induced hair follicle formation.^18^, ^36^ Nonetheless, they have limited clinical applications.^39^ Hair follicle neogenesis using adult wild hFDPc have been only achieved using DPS, being a high variable and inefficient system. Although it mimics the cells native environment, it presents several limitations, not only in terms of cell nourishment, but also matrix composition. Due to the fibroblast‐like nature of these cells, any culture system devoted to hFDPc must not only provide a 3D arrangement but also a suitable extracellular matrix to be remodelled.

To our knowledge, there is no available information in the literature related to hFDPc viability within the spheroid. Here, we proved that the percentage of viable cells in the outer sheath of the spheroid was very low, regardless of the culture condition. Considering that the size of the spheroids is below the diffusion limit,^9^, ^10^ these results evidenced that the system itself, and not culture conditions, was a hindrance to cell viability. This assumption was later supported by cell viability and proliferation when compared to those of FM (Figure 1E). Nonetheless, these viability values dropped as the number of encapsulated cells increased, which suggests that there is an imbalance in the cell/volume ratio at high cell concentrations. This phenomenon can be directly related to matrix rearrangement mediated by the cells within the first 48 h,^35^, ^40^ as human fibroblasts attach to the extracellular matrix by means of RGD cell adhesion motifs, and as the number of cells increases, the relative number of RGD motifs for cell attachment decrease and hence, cell viability as well.

The maintenance of hFDPc intrinsic properties within the DPS and FM was proven by the positive expression of well‐known pluripotency markers of hFDPc.^2^ Of special interest is the induction of Col IV expression,^41^, ^42^ which has not been previously reported for the DPS system. Although immunofluorescence results showed negligible expression of Col IV in the DPS (Figure 2H) as compared to the FM (Figure 2L), RT‐qPCR showed a higher Col IV gene expression for DPS (Figure 2Q). These differences may be explained by a time difference in the hFDPc mRNA and protein expression of Col IV within the two systems. On this basis, Col IV expression for FM occurs earlier, and at D_0_ it can be detected in the cell membrane, whereas for the same time point, DPS showed higher levels of endogenous mRNA levels undetectable through immunofluorescence. Moreover, high cell viability within FM allowed to characterize matrix remodelling mediated by cells 17 days after formation, with a strong positive expression of Col IV and Versican (Figure 2M–P). While both systems induce in vitro epidermal invagination and cell differentiation into hair follicle fate,^43^, ^44^, ^45^ FM proved to be a more reproducible and efficient system (50%), which can now be explained by the low viability values provided by DPS. Surprisingly, while platelet‐rich fibrin has recently shown its effectiveness in promoting hFDPc proliferation and migration,^25^ the presence of platelets have no effect neither in cell restoration capacity of its intrinsic properties nor hair follicle induction efficiency.

In conclusion, in this work, we proved that fibrin matrices succeed in cell restoring hFDPc intrinsic properties while boosting cell viability, but also ensure reproducibility and efficiency in the induction of epidermal invagination. These systems will allow not only the generation of complex in vitro skin models, but it is also a crucial step in the pursuit of human hair follicle regeneration therapies.

## AUTHOR CONTRIBUTIONS

Cristina Quílez, José Luis Jorcano, and Diego Velasco involved in conceptualization. Cristina Quílez performed the investigation, formal analysis, and writing—original draft. Leticia Valencia, Jorge González‐Rico, Leticia Suárez‐Cabrera, and Lidia Amigo‐Morán helped in methodology. Diego Velasco involved in reviewing and editing.

## CONFLICT OF INTEREST STATEMENT

The authors declare no conflict of interest.

## Supporting information


**DATA S1.** Supporting Information.

## Data Availability

All the data are available from the corresponding author Diego Velasco Bayón under reasonable request.
