# Supplementary material for: In vitro induction of hair follicle signatures using human dermal papilla cells encapsulated in fibrin microgels
Source: Cell Prolif. 2023 Aug 4;57(1):e13528. doi: 10.1111/cpr.13528 (PMC10771113; doi:10.1111/cpr.13528)
Supplement: Supplementary file 1 — DATA S1. Supporting Information. [file CPR-57-e13528-s001.docx]

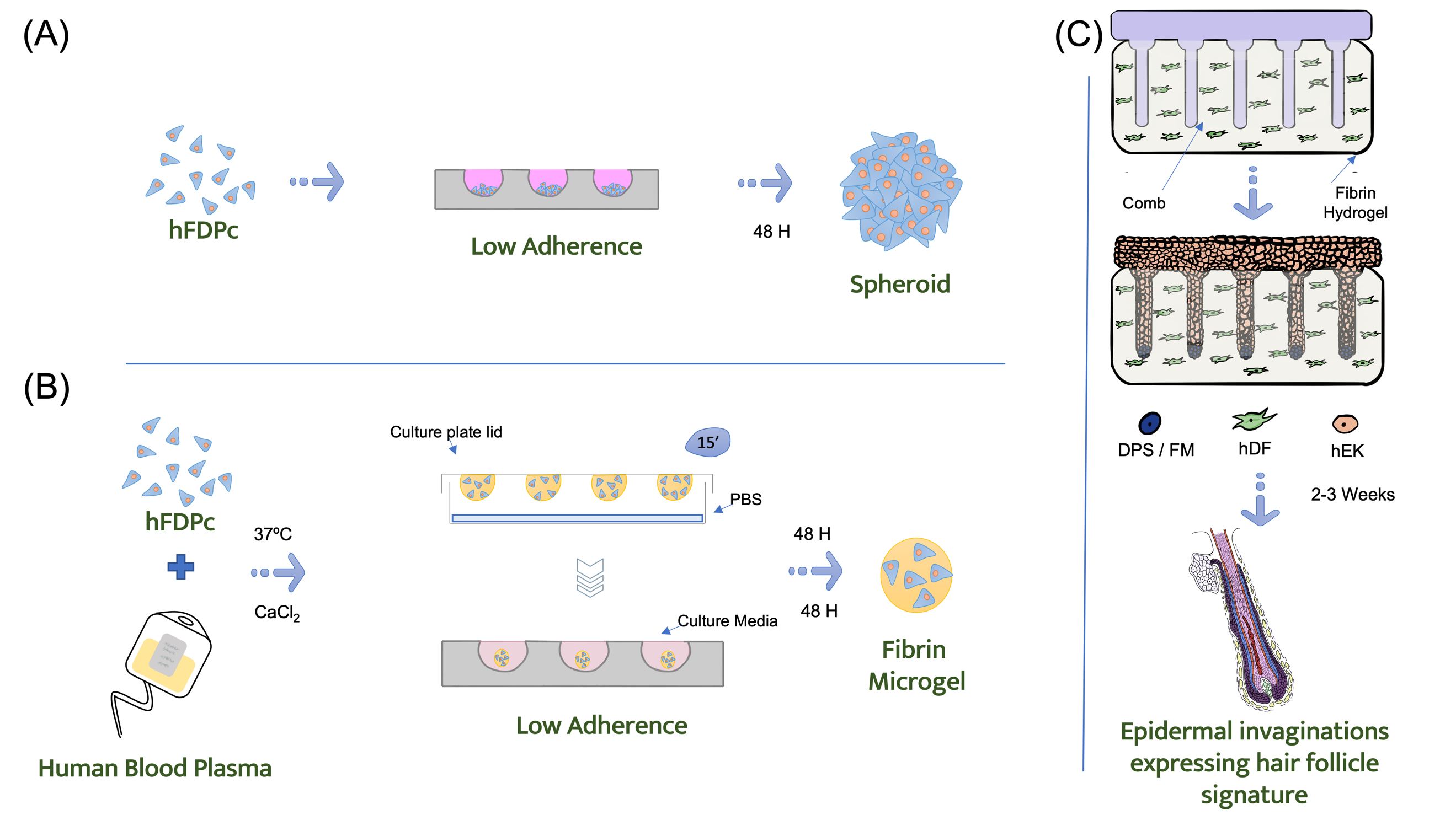


Figure S1: **Schematic representation of the experimental setting.** Methodology for the generation of (A) DPS and (B) FM. Experimental setting for the induction of hair follicle differentiation in *in vitro* skin equivalents using DPS and FM.

Text S1: Detailed description of the methods of the plasma-derived fibrin hydrogel preparation for FM and organotypic skin.

All hFDP cells, human primary fibroblasts and were purchased at PromoCell (PromoCell, Germany). Specific cell culture condition and culture media composition are described in detail elsewhere(1–4).

**Fibrin Microgels**

The volume of plasma to be used for the matrix to have a final fibrin concentration of 2.4mg/mL was calculated. Additionally, to avoid fibrin-clot degradation the solution was supplemented with the fibrinolytic agent, Amchafibrin (500mg, MedaPharma SL, Spain), to a final concentration of 0.016% (w/v). To activate the coagulation cascade, filtered CaCl_2_ 1% was added in a final concentration of 0.16 % (w/v). Finally, culture medium was added to adjust the desire final volume. For cell encapsulation, when hFDPc were at 80% confluency, culture plate was washed twice with PBS 1X and incubated with 1mL trypsin for 5 minutes at 37ºC and 5% CO_2_ for cell detachment. Trypsin was neutralized using 1mL culture media, from which 10μL cell suspension was used for cell counting in a Neubauer chamber, and the rest was centrifuged at 700 x g for 5 minutes. Supernatant was removed and cells were ready to proceed with microgel encapsulation. Once the volumes for all the components were known, FM were prepared as follows: first, cell pellet was resuspended and homogenized at the desired cell density using blood plasma (PPP/PRP) (see below). Secondly, the amount of plasma needed, previously calculated, was transferred to a 1.5mL Eppendorf tube. Then, Amchafibrin and culture medium were added to the cell-plasma suspension and homogenized. Finally, and just before drop generation, CaCl2 1% required volume was added to the working solution. For drop generation, 2μL drops of working solution were deposited with a micropipette onto the lid of a 35-mm culture plate (Figure S1B). When finished, the culture plate was filled with PBS 1X and covered with the lid containing the drops to avoid evaporation. Due to high cell concentration, plasma coagulation reaction rapidly occurred, reason why only 25 drops were generated at a time. To induce plasma coagulation culture plates were incubated for 15 minutes at 37ºC and 5% CO_2_. At this point, using a stereomicroscope and with the help of pipet tips, FM were detached from the lid of the culture plate and transferred to a LA-plate for spheroid culture with 100 μL of culture medium (Figure S1B) and cultured for 48 hours at 37ºC and 5% CO2 prior to use. FM were cultured in LA-plate instead (BIOFLOAT^TM^ 96-well plate (faCellitate, Germany)) of a usual p96-well plate because its U-shaped low-adhesive well avoided microgel adhesion to the border of the bottom plate and facilitated its manipulation.

**Organotypic skin**

Hydrogels of 4 mL are prepared as follows: for a known fibrin concentration, the volume of plasma needed to have a final fibrin concentration of 2.4 mg/mL was calculated and supplemented with the fibrinolytic agent, Amchafibrin, to a final concentration of 0.016% (w/v) to avoid fibrin-clot degradation. For coagulation cascade activation, filtered CaCl_2_ 1% was added in a final concentration of 0.16% (w/v) and then, human fibroblasts were incorporated at a cell density of 20.000 cells/mL diluted in 160μL culture medium. Final volume was adjusted using filtered NaCl 0.9%. Once homogenized, the solution was deposited within insert culture plates placed inside a 6-well plate, and the previously sterilized 3D printed comb with 10 tooth/ cm^2^ (Figure S2A) was placed on top of the insert for hydrogel polymerization (Figure S2B, C). One hour after incubation at 37ºC and 5% CO_2_, the comb was carefully removed, and 2 mL of culture medium were added on top and below the insert and incubated for 24 hours to remove the excess of CaCl_2_. Culture medium was removed from plasma hydrogels, after which DPS/FM were introduced in the holes one by one with a micropipette and the help of a stereomicroscope (Figure S2E). When finished, the excess of culture medium was removed from the top of the hydrogel. For DPS/FM and epidermal keratinocytes co-culture, 2 · 10^6^ epidermal keratinocytes suspended in culture media were placed on top of each hydrogel to fill all the holes and cover the hydrogel surface. Extra culture media was placed in the bottom of the insert, and hydrogels were cultured for 48 hours at 37ºC and 5% CO_2_ for keratinocyte attachment. After that, culture medium was replaced by sufficient volume of culture media with low serum to cover the hydrogel surface and the lower part of the plate to promote cell differentiation but not proliferation after which they were cultured at 37ºC and 5% CO_2_ up to for 6-weeks. Epidermal differentiation requires hydrogel culture in the air-liquid interface, whereas hair follicle differentiation was induced in submerged culture. In that way, keratinocyte differentiation into a stratified epidermis was not promoted due to the absence of air but instead, hFDPc signaling within the DPS/FM induced keratinocyte differentiation into a hair follicle structure.


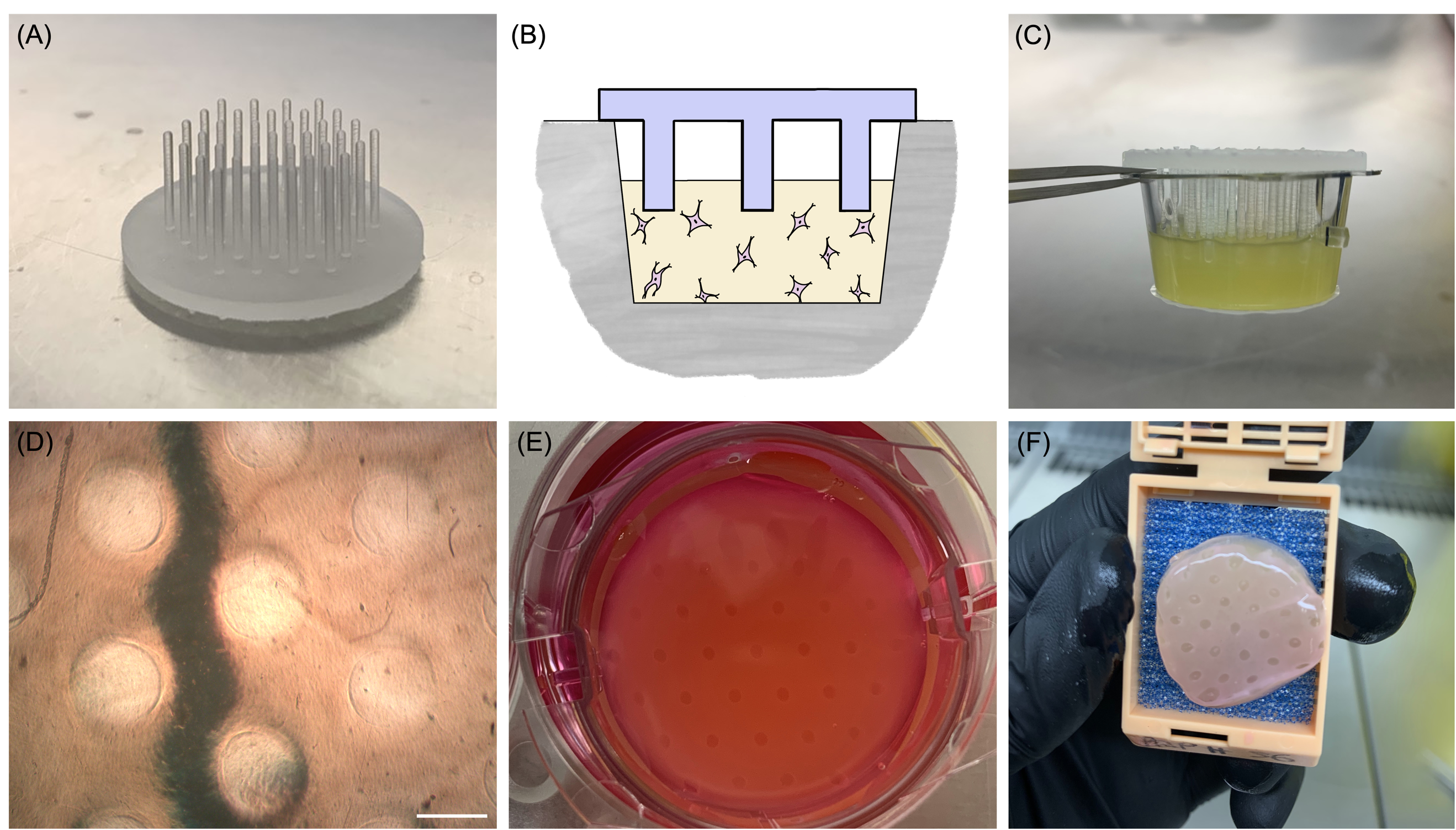


Figure S2: **Methodology for the induction of hair follicle differentiation using organotypic skin models**. Hole pattern formation for hair follicle generation in fibrin hydrogel: (A) 3D printed comb (B) Schematic representation of the experimental setting used for micropatterning in organotypic skin cultures. (C) Experimental setting for pattern generation in human plasma hydrogel and (D) pattern distribution on hydrogel surface for 20 tooth/cm^2^. Scale bar: 1mm. In (E), hole-pattern formation in human plasma hydrogels and DPS-hEK co-culture in plasma hydrogels for hair follicle differentiation and (F) preparation of human plasma hydrogels for histological processing after 3 weeks in culture.

Table S1: Antibodies for immunofluorescence analysis

| Antibody | Reference; Supplier | Dilution | Function/Structure |
| --- | --- | --- | --- |
| anti-BrdU | MA3-071, Thermo Fisher Scientific, USA | 1:100 | Cell Proliferation |
| Anti-AlkalinePhosphatase (ALP) | ab65834, Abcam, UK | 1:50 | hFDPc stemness (2) |
| Anti-Versican | PA1-1748A, Thermo Fisher Scientific, USA | 1:200 | hFDPc stemness & ECM (2,5) |
| Anti-α-Smooth Muscle Actin (αSMA) | DB147-0.1, DB Biotech, Slovakia | 1:100 | hFDPc stemness (2) |
| Anti-Collagen IV (Col IV) | 14-9871-82, eBiosciences^TM^, USA | 1:100 | hFDPc stemness & ECM (6) |
| anti-K15 | MA5-11344, Thermo Fisher Scientific, USA | 1:50 | Hair Follicle Bulge (7) |
| anti-K14 | MA5-11599, Thermo Fisher Scientific, USA | 1:200 | Hair Outer Root Sheath (7) |
| anti-K71 | PA5-28558, Thermo Fisher Scientific, USA | 1:200 | Hair Inner Root Sheath (8) |
| anti-K75 | ab254740, Abcam, UK | 1:50 | Hair Companion Layer (9) |
| anti-K31 | PA5-28522, Thermo Fisher Scientific, USA | 1:100 | Hair shaft (8) |

Text S2: Detailed description of the methods for the Real-time quantitative PCR

At D_0_, DPS and FM were collected for RNA extraction. hFDPc embedded within the fibrin gels were extracted using a fibrinolytic solution of 50FU/mL nattokinase (NSK-SD; Japan Bio Science Laboratory Co., Ltd) dissolved in PBS containing 1mM EDTA (Thermo Fisher Scientific, USA) (10). After PBS washing, FM were digested for 30 minutes at 37ºC and centrifuged to extract the cell pellet. Total RNA was extracted using miRNeasy Mini Kit (217504, Quiagen, Germany) following manufacturer’s instructions. 300ng of total RNA was subjected to reverse transcription using RevertAid H Minus FirstS trand cDNA Synthesis Kit (K1632, Thermo Fisher Scientific, USA). The reaction was carried out in a thermal cycler using the following conditions: 5 min at 25 ºC followed by 60 min at 42 ºC and finished with 5 min at 70 ºC to stop reaction. Real-time quantitative PCR was performed using GoTaq qPCR master mix (A600A, Promega, USA) and reaction was carried out in QuantStudio™ 6 equipment (Applied Biosystems, USA). Primer sequences to detect specific gene expression are listed on **Table S2**. YWHAZ gene was utilized as the endogenous control for normalization and 2^(−ΔΔCt)^ method was applied for relative quantification of gene expression (11).

Table S2: Primer used for real-time quantitative PCR

|  | | |  |
| --- | --- | --- | --- |
| **Gene name** | **Primer sequence (5' to 3')** | | **Amplicon size** |
| Versican | Fw: | CCAGCAAGCACAAAATTTCA | 158 |
|  | Rv: | TGCACTGGATCTGTTTCTTCA |  |
| ALP | Fw: | ATTGACCACGGGCACCAT | 57 |
|  | Rv: | CTCCACCGCCTCATGCA |  |
| α-SMA | Fw: | TGGCTATTCCTTCGTTACTACTGCT | 146 |
|  | Rv: | CATCAGGCAACTCGTAACTCTTCTC |  |
| Collagen IV | Fw: | TAGAGAGGAGCGAGATGTTC | 129 |
|  | Rv: | GTGACATTAGCTGAGTCAGG |  |
| YWHAZ | Fw: | ACTTTTGGTACATTGTGGCTTCAA | 94 |
|  | Rv: | CCGCCAGGACAAACCAGTAT |  |

Table S3: Comparative analysis of hFDPc viability (Live/Dead) and proliferation (BrDU) within DPS and FM for all culture conditions.

|  |  |  | Culture Conditions |  |
| --- | --- | --- | --- | --- |
|  |  | *Cell Number* | *DPs (%)* | *FM (%)* |
|  |  |  | *Static/Dynamic* |  |
|  |  | *6000* | *24.5/25* | *40.2* |
| *Viability (%)* | *Live/Dead®* | *3000* | 25/28 | 44.5 |
|  |  | *1500* | 25/19 | 50 |
|  |  | *750* | 23/20 | 85 |
| *Proliferation (%)* | *BrdU* | *3000* | 5 | 16.7 |


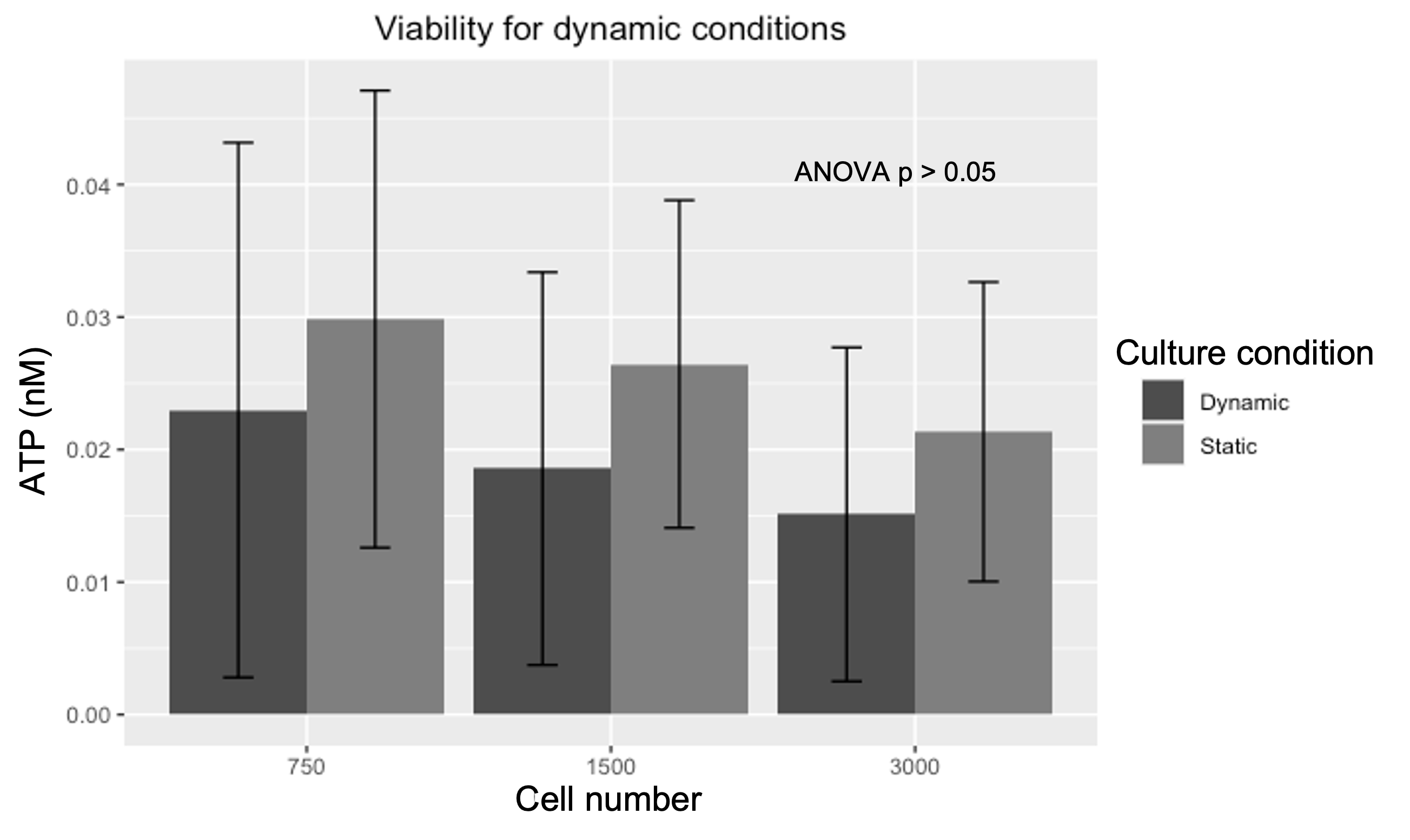


Figure S3: **Cell viability in terms of ATP activity.** Viability of hFDPc within DPS at day 0 in terms of ATP content (nM) for different cell number at static/dynamic conditions. Five spheroids from each condition were used to measure ATP concentration. Statistical significance: p-value < 0.05 *, p-value < 0.01 ** and p-value < 0.001 ***.


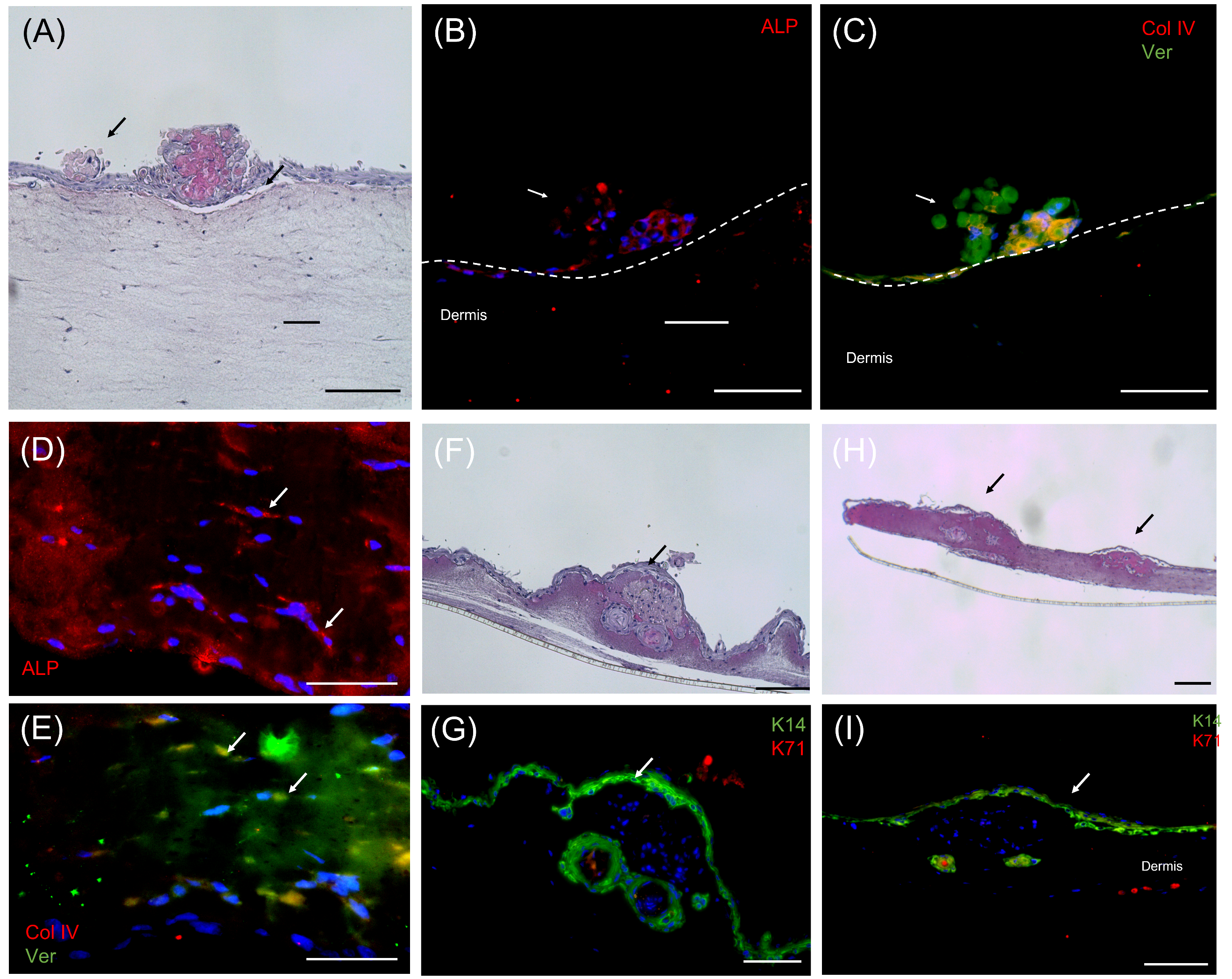


Figure S4: **Epidermal invaginations expressing hair-follicle signatures.** DPS on the epidermis of an organotypic skin (A) expressing ALP (B), Col IV and Versican (C) after three weeks in culture. Scale bar: 200µm. hFDPc within FM expressing ALP (D), Col IV and Versican (E) after three weeks in culture. Scale bar: 50 µm. Analysis of the tubular structures (F, H) expressing hair-specific keratins K14/K71 (G, I) induced in organotypic skin cultures by FM after three weeks in culture. White and black arrows point the FM in the dermal compartment of the construct. In blue, nuclei are stained with DAPI. Scale bar: 200µm.

## References

1. Montero A, Quílez C, Valencia L, Girón P, Jorcano JL, Velasco D. Effect of Fibrin Concentration on the In Vitro Production of Dermo-Epidermal Equivalents. Int J Mol Sci. 2021 Jun;22(13).

2. Higgins CA, Richardson GD, Ferdinando D, Westgate GE, Jahoda CAB. Modelling the hair follicle dermal papilla using spheroid cell cultures. Vol. 19, Experimental dermatology. Denmark; 2010. p. 546–8.

3. Meana A, Iglesias J, Del Rio M, Larcher F, Madrigal B, Fresno MF, et al. Large surface of cultured human epithelium obtained on a dermal matrix based on live fibroblast-containing fibrin gels. Burns. 1998;24(7):621–30.

4. Llames SG, Del Rio M, Larcher F, García E, Escamez MJ, Jorcano JL, et al. Human plasma as a dermal scaffold for the generation of a completely autologous bioengineered skin. Transplantation. 2004;77(3):350–5.

5. Soma T, Tajima M, Kishimoto J. Hair cycle-specific expression of versican in human hair follicles. J Dermatol Sci [Internet]. 2005 Sep 1;39(3):147–54. Available from: https://doi.org/10.1016/j.jdermsci.2005.03.010

6. Couchman JR. Rat Hair Follicle Dermal Papillae Have an Extracellular Matrix Containing Basement Membrane Components. J Invest Dermatol [Internet]. 1986;87(6):762–7. Available from: https://www.sciencedirect.com/science/article/pii/S0022202X8690182X

7. Langbein L, Schweizer J. Keratins of the human hair follicle. Int Rev Cytol. 2005;243:1–78.

8. Kiso M, Tanaka S, Saba R, Matsuda S, Shimizu A, Ohyama M, et al. The disruption of Sox21-mediated hair shaft cuticle differentiation causes cyclic alopecia in mice. Proc Natl Acad Sci U S A. 2009 Jun;106(23):9292–7.

9. Mesler AL, Veniaminova NA, Lull M V, Wong SY. Hair Follicle Terminal Differentiation Is Orchestrated by Distinct Early and Late Matrix Progenitors. Cell Rep. 2017 Apr;19(4):809–21.

10. Carrion B, Janson IA, Kong YP, Putnam AJ. A safe and efficient method to retrieve mesenchymal stem cells from three-dimensional fibrin gels. Tissue Eng Part C Methods. 2014 Mar;20(3):252–63.

11. Livak KJ, Schmittgen TD. Analysis of relative gene expression data using real-time quantitative PCR and the 2(-Delta Delta C(T)) Method. Methods. 2001 Dec;25(4):402–8.
